# Does Presentation Format Influence Visual Size Discrimination in Tufted Capuchin Monkeys (*Sapajus* spp.)?

**DOI:** 10.1371/journal.pone.0126001

**Published:** 2015-04-30

**Authors:** Valentina Truppa, Paola Carducci, Cinzia Trapanese, Daniel Hanus

**Affiliations:** 1 Institute of Cognitive Sciences and Technologies, National Research Council (CNR), Rome, Italy; 2 Department of Biology, University of Rome Tor Vergata, Rome, Italy; 3 Department of Developmental and Comparative Psychology, Max Planck Institute for Evolutionary Anthropology, Leipzig, Germany; University of Padova, ITALY

## Abstract

Most experimental paradigms to study visual cognition in humans and non-human species are based on discrimination tasks involving the choice between two or more visual stimuli. To this end, different types of stimuli and procedures for stimuli presentation are used, which highlights the necessity to compare data obtained with different methods. The present study assessed whether, and to what extent, capuchin monkeys’ ability to solve a size discrimination problem is influenced by the type of procedure used to present the problem. Capuchins’ ability to generalise knowledge across different tasks was also evaluated. We trained eight adult tufted capuchin monkeys to select the larger of two stimuli of the same shape and different sizes by using pairs of food items (Experiment 1), computer images (Experiment 1) and objects (Experiment 2). Our results indicated that monkeys achieved the learning criterion faster with food stimuli compared to both images and objects. They also required consistently fewer trials with objects than with images. Moreover, female capuchins had higher levels of acquisition accuracy with food stimuli than with images. Finally, capuchins did not immediately transfer the solution of the problem acquired in one task condition to the other conditions. Overall, these findings suggest that – even in relatively simple visual discrimination problems where a single perceptual dimension (i.e., size) has to be judged – learning speed strongly depends on the mode of presentation.

## Introduction

Many comparative studies concerning learning abilities in the visual cognition domain focus on non-human primates. Like humans, all other diurnal primate species mainly rely on sight to gather information from the environment [1, 2]. Moreover, these species have visual systems that share many properties with that of human beings [3–5].

Virtually all experimental paradigms employed to study visual cognition in humans and non-human species are based on discrimination tasks involving the choice between two or more visual stimuli. For this purpose, different types of stimuli and stimulus presentations can be used. The necessity to carry out in-depth analyses of cognitive processes has led to the development of increasingly sophisticated methods for data collection. The last few decades in particular have seen widespread use of computerised procedures in an increasing number of taxonomic groups, such as apes [6–9], Old World monkeys [10–13], New World monkeys [14–22], dogs [23, 24], horses [25], pigeons [26–33], corvids [34], and tortoises [35]. Despite this trend, little is known about the generalisability of such computerised methods to more natural settings involving ‘real’ three-dimensional stimuli (e.g., food). This highlights the necessity to compare data obtained with different methodological approaches, e.g., computerised procedures and procedures involving a human experimenter [36–39].

Computerised procedures present both advantages and limits when compared with non-computerised methods. On the one hand, computerised tasks allow the administration of a higher number of trials within a scheduled time slot and also the presentation of stimuli and registration of responses very precisely (in terms of both accuracy and response time). Moreover, computerised procedures can allow to completely prevent experimenter biases. On the other hand, these types of procedures are almost exclusively administered by using images presented on a computer screen, with only very few exceptions involving, for example, computerised procedures where individuals are required to manipulate mechatronic objects [40] or to manipulate a joystick device controlling a projected laser dot in order to select food items [41].

Besides the type of experimental procedure used for stimuli presentation, the type of stimuli *per se* might also consistently affect subjects’ behaviour. There is evidence, for example, that the use of food as a stimulus to be discriminated in cognitively demanding choice tasks might positively [42] or negatively [43] influence capuchin monkeys’ performance according to task requirements. On the one hand, Addessi et al. [42] found that capuchins were significantly more accurate in quantity discrimination judgements with food items than with tokens (i.e., inherently non-valuable objects that acquire an associative value upon exchange with the experimenter, [44]). On the other hand, in a reverse-reward contingency task, where capuchins had to select the smaller option to receive the larger reward, their performance was significantly lower with food items than with tokens [43]. In addition, Boysen et al. [45] showed that when chimpanzees were required to solve a reverse-reward contingency task, four out of five individuals preferred to choose the larger option despite it meaning they received the smaller amount of food; moreover, they chose the option including the larger food item independently of the total amount of food items presented by the two options. Overall, findings on capuchins and chimpanzees suggest that the particular ecological salience of food may induce a powerful predisposition to select the option offering the larger amount of items very accurately and that this accuracy can also be biased by the size of individual food items.

Thus, systematic comparisons of the same subjects tested by using different types of methods (i.e., within-subjects experiments) would contribute to clarifying how methodological aspects may affect visual perceptual learning in a given task. Moreover, comparisons across different tasks in which the same visual discrimination problem is presented, also represent an opportunity to test the ability to use similarities to transfer the solution of a familiar problem across novel but functionally equivalent conditions.

The ability to understand similarities and analogies is a fundamental aspect of advanced cognition [46, 47]. Evidence of the ability to use an identity concept has been reported in several non-human species belonging to very different taxonomic groups (chimpanzees [48, 49]; baboons [50, 51]; macaques [12, 52, 53]; capuchin monkeys [21, 22, 54]; dolphins [55–57]; sea lions [58, 59]; rats [60]; parrots [61]; jackdaws [62]; pigeons [28, 63–65]). More recently, an increasing amount of data has also demonstrated that non-human primates other than apes are able under specific training conditions to solve analogical problems involving the ability to understand second-order relations, i.e., relations between relations [15, 66–68]. Usually, studies to determine conditions under which this type of ability occurs are carried out presenting preliminary discrimination training followed by a testing phase in which completely novel stimuli are presented. In fact, only if a relationship between stimuli is coded in an abstract rule/concept can it be applied to new stimuli never seen before [69]. Standard experimental protocols basically rely on the presentation of novel stimuli in a task in which all other problem features are the same to guarantee a constant ‘framework of reference’ for the subject.

Conversely, studies in which the same type of problem was presented in different frameworks have shown only limited evidence of the ability of non-human species to generalise across conditions. For example, Martin-Ordas and Call [70] found that apes trained to solve functionally equivalent tool-using tasks that require them to overcome obstacles to get a reward (i.e., trap tasks), showed some knowledge about the effects that traps have on slow-moving unsupported objects. However, this knowledge did not seem to be abstract enough to allow them to establish general analogies between perceptually dissimilar tasks. The authors argued that perceptual components like different spatial arrangements of the tasks could not be perceived as functionally equivalent by the apes and therefore could have generated some novelty effect.

In the present study, we assessed whether visual learning ability of capuchin monkeys varies on the basis of both the type of stimuli and the type of procedure used. For this purpose, we evaluated learning speed and response accuracy of capuchin monkeys faced with different versions of the same visual discrimination problem, i.e., to choose the larger stimulus between two stimuli of the same shape. First, we compared two experimental conditions that can be considered to be the most different from each other: the discrimination of food items (that could be directly obtained and manipulated after the choice) and the discrimination of images presented on a computer screen. Next, we assessed monkeys’ ability to discriminate between non-edible objects. This allowed us to both evaluate learning processes to achieve acquisition in each condition and to evaluate monkeys’ generalisation ability across different conditions.

### Experiment 1: Discrimination of food items and computer images

Two conditions were included in Experiment 1: (1) the *Food* condition, in which the stimuli to discriminate between were pairs of food items presented on a table, and (2) the *Image* condition, in which the stimuli were pairs of figures presented on a computer screen. The order of the presentation of the conditions was counterbalanced across subjects. Half of the monkeys were tested in the *Image* condition after having completed the *Food* condition, whereas the other half received the *Image* condition first. We hypothesised that the monkeys would succeed in achieving the learning criterion with both foods and images, although we expected them to need fewer trials and to be more accurate in the *Food* compared to the *Image* condition. Additionally, we expected that they would take a similar number of training days to achieve the criterion in both conditions since a higher number of daily trials could be administered in the computerised procedure.

## Method

### Subjects

The subjects were eight tufted capuchin monkeys derived from individuals of different provenience and considered to be unknown combinations of species of the genus *Sapajus*. Recent data have revealed that capuchin monkeys, traditionally identified as the single genus *Cebus*, are two genera: (i) the robust (tufted) forms are now classified as the genus *Sapajus*, and (ii) the gracile (untufted) forms are retained as the genus *Cebus* [71, 72]. The sample included four males (Robot, Sandokan, Pedro and Robin Hood) and four females (Roberta, Robiola, Rucola and Quincy). All subjects were adults (12–27 years old) born in captivity. Capuchins were hosted at the Primate Center of the Institute of Cognitive Sciences and Technologies, CNR, Rome, Italy. They belonged to three groups, each housed in an indoor–outdoor enclosure (indoor: 5 m^2^ × 2.5 m high; outdoor: 40–130 m^2^ × 3 m high). Each indoor enclosure included two large cages, an experimental box and an area for the experimenter. Capuchins were individually tested in the experimental box (180 cm × 75 cm × 75 cm; the box floor is 80 cm higher than the floor of the rest of the room) to which they had access through a sliding door from the adjacent indoor cage. Each subject was separated from the group just before the daily testing session solely for the purpose of testing. Testing occurred between 9:30 a.m. and 3:15 p.m. Water was freely available at all times. Fresh fruit, vegetables and monkey chow were provided in the afternoon after testing. In the *Food* condition, half of the monkeys (Roberta, Robin Hood, Sandokan, Pedro) were already familiar with the experimental apparatus because they took part in a precedent experiment on tool choice where the same apparatus was used [73]. The other monkeys were familiarised to with the apparatus immediately before starting this experiment. In the *Image* condition, all the subjects were already familiar with the computerised workstation because they took part in a training programme with computerised matching-to-sample tasks [14–16]. None of the subjects had been tested with a computerised two-alternative choice task before.

### Apparatus

#### Food condition

**Fig 1:**
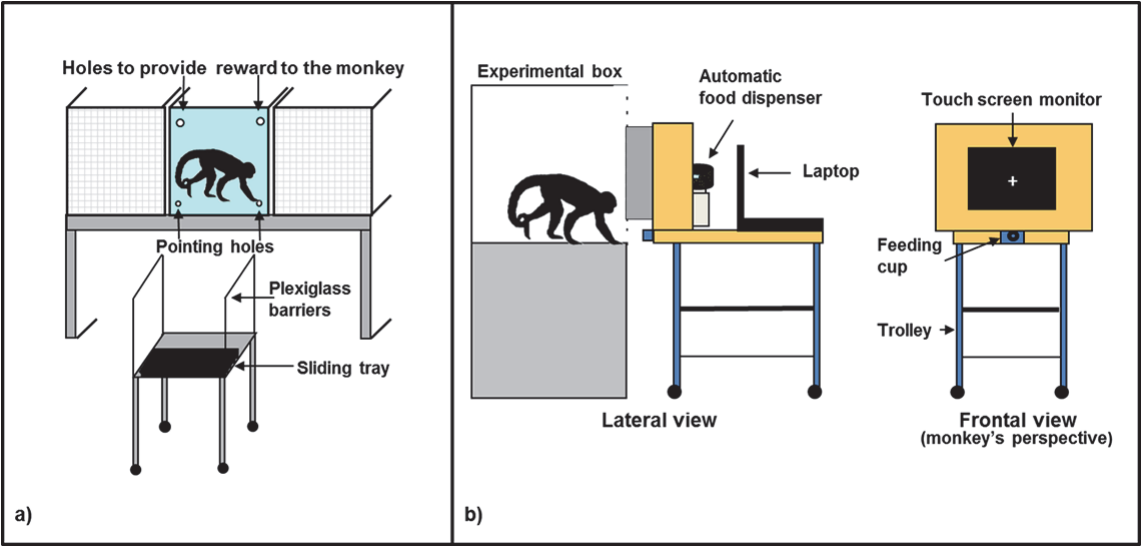
Experimental apparatuses. (a) Experimental apparatus used in the *Food* condition of Experiment 1 and in the *Object* condition of Experiment 2; (b) Experimental apparatus used in the *Image* condition of Experiment 1.

The apparatus consisted of a metal trolley (59 cm long, 64 cm wide, 92 cm high) with a sliding tray (54 cm × 20 cm), which could be moved forward and backward on a wooden support (59 cm × 56 cm) and two Plexiglas barriers (51 cm × 53 cm) on the lateral sides (Fig 1A). The apparatus was installed in front of the experimental box (approximately at a distance of 6 cm), behind a transparent Plexiglas panel (56 cm × 73 cm) mounted on the front wall of the experimental box. The transparent panel had four holes, situated at the corners: Two small ones (1.5 cm diameter) at the bottom for ‘pointing’ actions (i.e., to insert a finger into a hole spatially matching the selected stimulus) and two larger ones (3.5 cm diameter) at the top through which the monkey received the reward. Stimuli were located on the sliding tray and were gradually moved closer to the subject, while the monkey was positioned in front of the apparatus looking at the stimuli.

#### Image condition

The apparatus consisted of a laptop (model Acer Aspire 1400 Series) connected to a 17” touch screen (model Tyco Electronics, ET1729L-7UEA-1-D-GY-G) and an automatic food dispenser (model ENV-203-45, MED Associates, Inc. Georgia, VT) (Fig 1B). The E-Prime software (Psychology Software Tools, Inc.) was used as the stimulus generator and served both to present the stimuli and to record the response behaviour. The food dispenser was programmed to deliver one 45-mg banana-flavoured pellet (TestDiet, Richmond, IN, USA) when the monkey provided a correct response during the experimental trial. The pellet was delivered into a PVC feeding cup (with a diameter of 4 cm) located 14.5 cm below the touch screen in the centre. A wooden frame (58 cm wide × 54 cm high) with a central aperture (36 cm wide × 30 cm high) surrounded the touch screen. The food dispenser was placed behind the wooden frame, out of sight of the subject. The laptop was placed at the back of the touch screen to allow the experimenter to follow the progress of the session. The touch screen, the food dispenser and the laptop were mounted on the top shelf of a trolley (90 cm long × 58 cm wide × 85 cm high). The apparatus was fastened to a metal panel (56.5 cm wide × 74 cm high) mounted on the front wall of the experimental box (Fig 1B). The metal panel had a central rectangular aperture (37.5 cm wide × 30 cm high) which corresponded spatially to the touch screen position.

### Stimuli

#### Food condition

**Fig 2:**
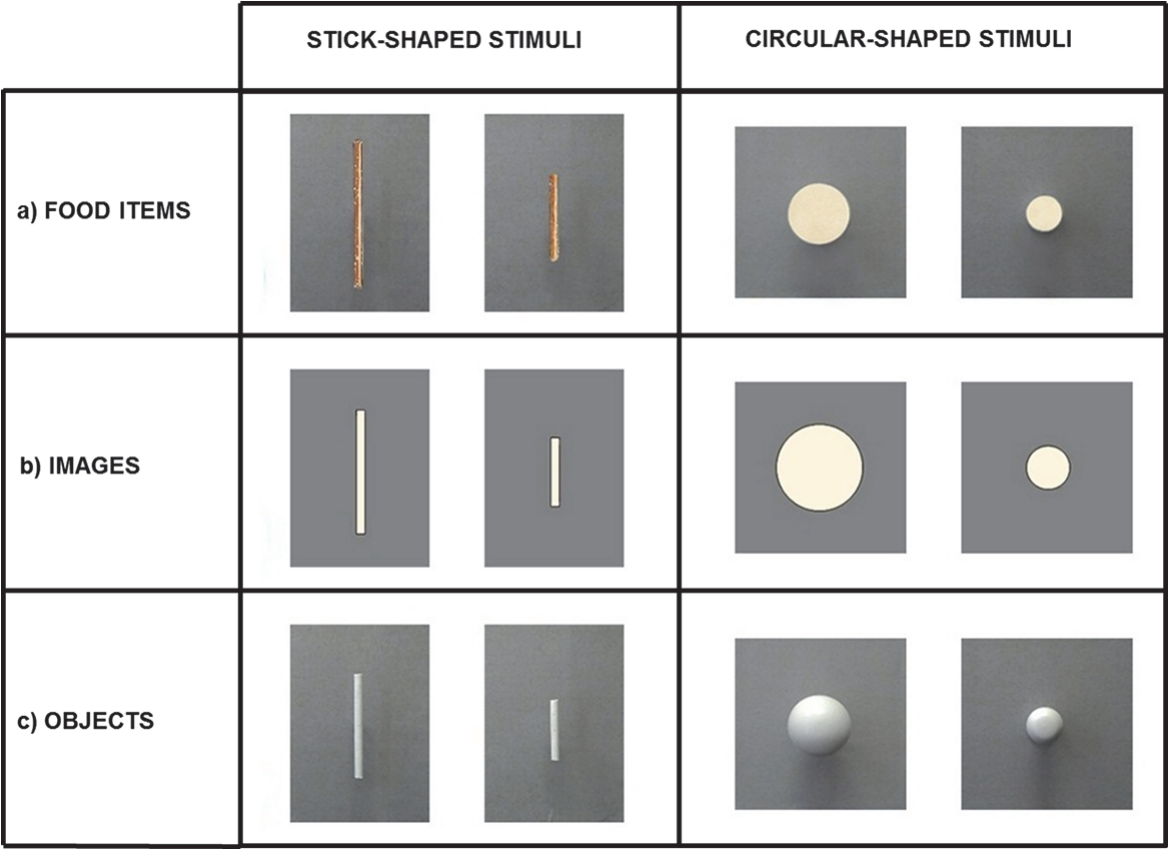
Stimulus sets. (a) Stimuli used in the *Food* condition of Experiment 1 were a pair of salted sticks of different lengths and a pair of wafers of different diameters; (b) Stimuli used in the *Image* condition of Experiment 1 were a pair of lines of different lengths and a pair of circles of different diameters; (c) Stimuli used in the *Object* condition of Experiment 2 were a pair of wooden sticks of different lengths and a pair of wooden spheres of different diameters.

The stimuli were two pairs of food items (Fig 2A), inserted into grey PVC (polyvinyl chloride) boards, held up vertically (approximately 45°) by a back support. The first pair of stimuli included two salted sticks (0.5 cm diameter) placed into PVC boards (14 cm × 20 cm): One stick was 10 cm high and the other was 6 cm high. The second pair included circular wafers placed into PVC boards (18 cm × 18 cm): One wafer had a 6.5 cm diameter and the other had a 3.7 cm diameter. The backs of the wafers had a little sugar glaze spread on them, in order to make them tastier for the monkeys.

#### Image condition

The stimuli were two pairs of white computer images presented in grey frames (Fig 2B). The first pair consisted of vertical lines (0.4 cm wide): One line was 4.5 cm high and the other was 2.5 cm high. The second pair consisted of circles: One circle had a 2.8 cm diameter and the other had a 1.5 cm diameter. Lines were presented within a 9.5 cm × 4.5 cm grey frame and circles were presented within a 9.5 cm × 7.5 cm grey frame.

### Procedure

#### Food condition

In each trial, the experimenter inserted the food items into the PVC boards and placed the stimuli on the sliding tray with the frontal part out of the monkey’s view. Only when the monkey was facing towards the apparatus were the stimuli simultaneously rotated to show the food items to the subject, with the sliding tray being moved forward into the monkey’s reach. The monkey made its choice by inserting a finger into one of the two small holes; the experimenter then handed the chosen food item to the monkey through the corresponding large hole towards the top of the panel. As soon as the monkey finished eating the food item and approached the apparatus again, a new trial started. Each monkey received one session of 16 trials per day: Half of the trials included pairs of salted sticks and the other half included pairs of wafers. Each larger stimulus was presented an equal number of times in both the right and the left position. Trials were presented in a random order. Sessions were administered 5 days per week. The learning criterion was achieved when monkeys chose the larger food item of each pair at least 7 out of 8 times (87.5%, binomial *p* <. 032) during three consecutive sessions.

#### Image condition

At the beginning of each trial, a white cross composed of two segments (0.8 cm high and 0.2 cm wide) appeared in the centre of the screen on a black background; when the monkey touched the cross two stimuli were simultaneously displayed. The initial touch of the central cross was adopted to ensure that monkeys were looking at the screen when the stimuli appeared. The subject had to indicate its choice by touching one of the two stimuli on the screen; the computer automatically recorded the choice. After the response, the display was immediately extinguished and, if the subject selected the larger stimulus, a food pellet was delivered. A correct response was followed by a 5-s inter-trial interval (ITI), whereas an incorrect response was followed by both a 10-s time out (TO) and a 5-s ITI. During the experimental trials and the ITI, the screen was light grey; during the TO, the screen was green. Each monkey received 64 trials per day (four sessions of 16 trials): Half of the trials included pairs of lines and the other half included pairs of circles. Each larger stimulus was presented an equal number of times in both the right and the left position. The trials were presented in a random order. Sessions were administered 5 days per week. The learning criterion was achieved when monkeys chose the larger image of each pair at least 7 out of 8 times (87.5%) during three consecutive sessions.

We presented a larger number of trials per day in the *Image* condition for two reasons: (1) to replicate procedures that usually featured computerised tasks, in which many trials separated by relatively short time intervals are administered and (2) to equalise the daily exposure time between the *Food* and the *Image* condition (i.e., about 20 minutes dealing with the task).

### Data analyses

The measures considered for analysis were the number of trials, the number of days and the accuracy scores to achieve the learning criterion (individual data are reported in S1 Table). Accuracy scores were defined as the average percentage of correct responses during the last three training sessions. Because the Kolmogorov-Smirnov test showed that the distribution of data did not significantly deviate from normality, we used parametric statistics to compare the mean scores between different conditions. Mixed-model analyses of variance (ANOVAs) were performed with Task Condition (image/computerised *vs*. food/not computerised) and Stimulus Shape (sticks/lines *vs*. wafers/circles) as within-subjects factors, and Order of Presentation of the task and Sex as between-subjects factors. Separate analyses were performed on the number of trials, the number of days and the accuracy scores to achieve the learning criterion respectively. Statistical significance was set at *p* ≤ 0.05, apart from with analyses that required correction for multiple tests (i.e., Bonferroni correction).

## Ethics Statement

This study and the research protocols used in Experiments 1 and 2 were approved by the Italian Health Ministry (Central Direction for the Veterinary Service, approvals n. 11/2011-C and n. 132/2014-C to V. Truppa). Housing conditions and experimental procedures were in full accordance with European law on humane care and use of laboratory animals and complied with the recommendations of the Weatherall Report [74]. To increase three-dimensional space available to the animals, indoor enclosures were furnished with perches and ropes and outdoor enclosures were furnished with logs, branches and ropes. Moreover, the presence of natural substrates, including woodchips on the ground, served to promote the monkeys’ exploratory behaviours. All subjects were habituated to the experimental cage, the experimental routine and the experimenters.

## Results

### Number of trials

**Fig 3:**
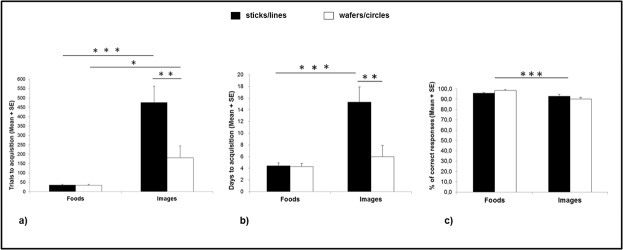
Monkeys’ performance in Experiment 1. Monkeys’ performance in the *Food* and *Image* conditions as a function of stimulus shape (sticks/lines and wafers/circles) was reported considering: (a) the number of trials to achieve the learning criterion (mean ± SE); (b) the number of days to achieve the learning criterion (mean ± SE); and (c) the percentage of correct responses in the last three training sessions (mean ± SE). *p <. 05; **p <. 01; ***p <. 001.

Significant main effects of both Task Condition [food: *M* = 34.5, *SE* = 3.7; image: *M* = 327.5, *SE* = 72.9; *F*_(1,4)_ = 13.2, *p* =. 022, *η* ^*2*^ *p* =. 767] and Stimulus Shape [sticks/lines: *M* = 255.0, *SE* = 43.3; wafers/circles: *M* = 107.0, *SE* = 31.4; *F*_(1,4)_ = 81.6, *p* =. 0008, *η* ^*2*^ *p* =. 953] were found. Moreover, the analysis revealed a significant interaction between Task Condition and Stimulus Shape [*F*_(1,4)_ = 82.9, *p* =. 0008, *η* ^*2*^ *p* =. 954] (Fig 3A). Post hoc analyses (Bonferroni test) across task conditions indicated that the number of learning trials with the salted sticks (*M* = 35, *SE* = 3.7) was significantly smaller than the number of learning trials with the lines (*M* = 475, *SE* = 86.9) (*p* =. 0002); similarly, the number of trials required with the wafers (*M* = 34, *SE* = 4.2) was significantly smaller than that required with the circles (*M* = 180, *SE* = 63.8) (*p* =. 018). Post hoc analyses across stimulus shapes revealed no significant difference in the number of learning trials between salted sticks (*M* = 35, *SE* = 3.7) and wafers (*M* = 34, *SE* = 4.2) in the *Food* condition (*p* = 1.0); whereas, in the *Image* condition, the number of learning trials for lines (*M* = 475, *SE* = 86.9) was significantly greater than for circles (*M* = 180, *SE* = 63.8) (*p* =. 001). No other main effects or interactions turned out to be significant.

### Number of days

A significant main effect of Stimulus Shape [sticks/lines: *M* = 9.8, *SE* = 1.3; wafers/circles: *M* = 5.1, *SE* = 0.9; *F*_(1,4)_ = 89.3, *p* =. 0007, *η* ^*2*^ *p* =. 957] and a Task Condition x Stimulus Shape interaction [*F*_(1,4)_ = 71.1, *p* =. 001, *η* ^*2*^ *p* =. 947] were found (Fig 3B). Post hoc analyses across task conditions revealed that the number of learning days with the salted sticks (*M* = 4.4, *SE* = 0.5) was significantly smaller than the number of learning days with the lines (*M* = 15.3, *SE* = 2.6) (*p* =. 0008); whereas the number of learning days for wafers (*M* = 4.3, *SE* = 0.5) and that for circles (*M* = 6.0, *SE* = 1.9) were not significantly different (*p* =. 505). Moreover, post hoc analyses across stimulus shapes revealed no significant difference in the number of learning days between the salted sticks (*M* = 4.4, *SE* = 0.5) and the wafers (*M* = 4.3, *SE* = 0.5) in the *Food* condition (*p* = 1.0); whereas, in the *Image* condition, the number of learning days for lines (*M* = 15.3, *SE* = 2.6) was significantly greater than for circles (*M* = 6.0, *SE* = 1.9) (*p* =. 001). No other main effects or interactions turned out to be significant.

### Accuracy scores

A significant main effect of Task Condition [food: *M* = 97.1%, *SE* = 0.7; image: *M* = 91.4%, *SE* = 1.1; *F*_(1,4)_ = 80.5, *p* =. 0008, *η* ^*2*^ *p* =. 846] and a Task Condition x Sex interaction [*F*_(1,4)_ = 10.6, *p* =. 031, *η* ^*2*^ *p* =. 727] were found (Fig 3C). Post hoc analyses revealed that female capuchins had higher accuracy scores in the *Food* condition (*M* = 96.9%, *SE* = 0.7) than in the *Image* condition (*M* = 89.1%, *SE* = 0.8) (*p* =. 006); while males’ accuracy scores in the *Food* condition (*M* = 97.4%, *SE* = 1.1) did not significantly differ from accuracy scores in the *Image* condition (*M* = 93.8%, *SE* = 2.1) (*p* =. 094). No other main effects or interactions turned out to be significant.

### Transfer across tasks

To evaluate subjects’ immediate ability to generalise their discrimination performance across conditions, we analysed the percentage of correct responses at the beginning of their second-order condition (*Food* or *Image*). The four subjects that received the *Image* condition second did not achieve the learning criterion within the first three sessions (trials 1–48; Lines: Robot 41.67%, Robin Hood 45.83%, Roberta 50.0%, Robiola 50.0%; Circles: Robot 83.33%, Robin Hood 75.0%, Roberta 58.33%, Robiola 50.0%). Specifically, subjects that received the *Image* condition second were equally accurate in discriminating circles compared to subjects that received the *Image* condition first [*Image* second: *M* = 66.65%, *SE* = 7.60; *Image* first: *M* = 64.57%, *SE* = 2.69; *t*(6) = 0.26, *p* =. 806]. Furthermore, subjects that received the *Image* condition second were even significantly less accurate in discriminating the lines than subjects that received the *Image* condition first, again considering only the first three sessions [*Image* second: *M* = 46.87%, *SE* = 1.99; *Image* first: *M* = 63.55%, *SE* = 2.01; *t*(6) = -5.90, *p* =. 001] (Bonferroni correction for multiple tests, *p* =. 025).

On the other hand, two out of the four subjects that received the *Food* condition as the second task achieved the learning criterion within the first three sessions in discriminating between sticks (Pedro 92%) and between wafers (Sandokan 100%) respectively. However, it should be mentioned that two out of the four subjects that received the *Food* condition first also successfully discriminated between stimuli during the first three sessions (Sticks: Robot 100%, Robin Hood 96%; Wafers: Robot 100%, Robin Hood 100%). Thus, although some of the subjects that received the *Food* condition second seemed to exhibit an immediate transfer of performance from the *Image* to the *Food* condition, comparable performances were observed in subjects that received the *Food* condition first.

### Experiment 2: Discrimination of objects

The second experiment was carried out to clarify whether the better performance in the *Food* compared to the *Image* condition found in Experiment 1 was due to the use of edible items as stimuli for discrimination, or due to the influence of other characteristics of the non-computerised task (e.g., concrete 3D stimuli) which might have improved monkeys’ performance. For this purpose, monkeys were required to discriminate between pairs of (non-edible) objects of the same shape presented with the same apparatus used for the food items in Experiment 1.

## Method

### Subjects

The subjects were seven capuchin monkeys, the same tested in Experiment 1, except for one female, Rucola. We did not test her because she exhibited alarm vocalisations and threat reactions towards the objects used in this experiment, both behaviours that in this species are usually associated with the presence of dangerous stimuli [75]. All the monkeys were tested in Experiment 2 after they had completed Experiment 1. Since the *Object* condition was presented to all experimental subjects as the third task, we also presented this condition to a control group of capuchins that did not take part in Experiment 1, namely four adult capuchin monkeys, two females (Carlotta and Paprica) and two males (Paté and Vispo), with experimental histories comparable to those of the individuals tested in Experiment 1. This comparison between the experimental and control group allowed us to evaluate the occurrence of potential facilitation effects due to previous experience.

### Apparatus

The apparatus and the Plexiglas panel were the same as those used in the *Food* condition of Experiment 1 (Fig 1A).

### Stimuli

The stimuli were two pairs of white wooden objects, mounted on grey wooden boards, held up vertically by a back support (Fig 2C). The first pair of stimuli included two cylindrical wooden rods (0.8 cm diameter), each mounted on a wooden board (14 cm × 20 cm): One rod was 10 cm high and the other was 6 cm high. The second pair included two wooden spheres, each mounted on a wooden board (18 cm × 18 cm): One sphere had a diameter of 6.2 cm, and the other 3.7 cm.

### Procedure

At the beginning of each trial, the experimenter placed the wooden boards on the sliding tray with the frontal part out of the monkey’s view. Only when the monkey was facing towards the apparatus were the stimuli simultaneously rotated to show the objects to the subject, with the sliding tray being moved forward to get the stimuli into the subject’s reach. The monkey made its choice by inserting a finger into one of the two small holes of the Plexiglas panel. Whenever the monkey chose the larger stimulus, the experimenter handed over a piece of peanut through the corresponding large hole towards the top of the panel. As soon as the subjects finished eating the reward and approached the apparatus again, a new trial started. Each monkey received one session of 16 trials per day: Half of the trials included pairs of rods and the other half included pairs of spheres. Each larger stimulus was presented an equal number of times in the right and the left position. The trials were presented in a random order. Sessions were administered 5 days per week. The learning criterion was achieved when monkeys chose the larger object of each pair at least 7 out of 8 times (87.5%) during three consecutive sessions.

### Data analyses

The measures considered for analysis were the number of trials and the percentage of correct responses to achieve the learning criterion with objects (individual data are reported in S2 Table). Because the Kolmogorov-Smirnov test showed that the distribution of data did not deviate from normality, we used parametric statistics to compare the mean scores between different conditions. We performed mixed-model ANOVAs on the two measures considered, with Stimulus Shape (rods and spheres) as a within-subjects factor and Sex as a between-subjects factor. Moreover, we used dependent *t*-tests to compare performance on objects with performance on foods and images, both in terms of learning speed (number of trials to criterion) and percentage of correct responses to acquisition. Finally, experimental and control subjects were compared both in terms of learning speed and response accuracy to acquisition by using mixed-model ANOVAs with Stimulus Shape (rods and spheres) as a within-subjects factor and Group (Experimental and Control) as a between-subjects factor.

Statistical significance was set at *p* ≤ 0.05, apart from with analyses that required correction for multiple tests (i.e., Bonferroni correction).

## Results

**Fig 4:**
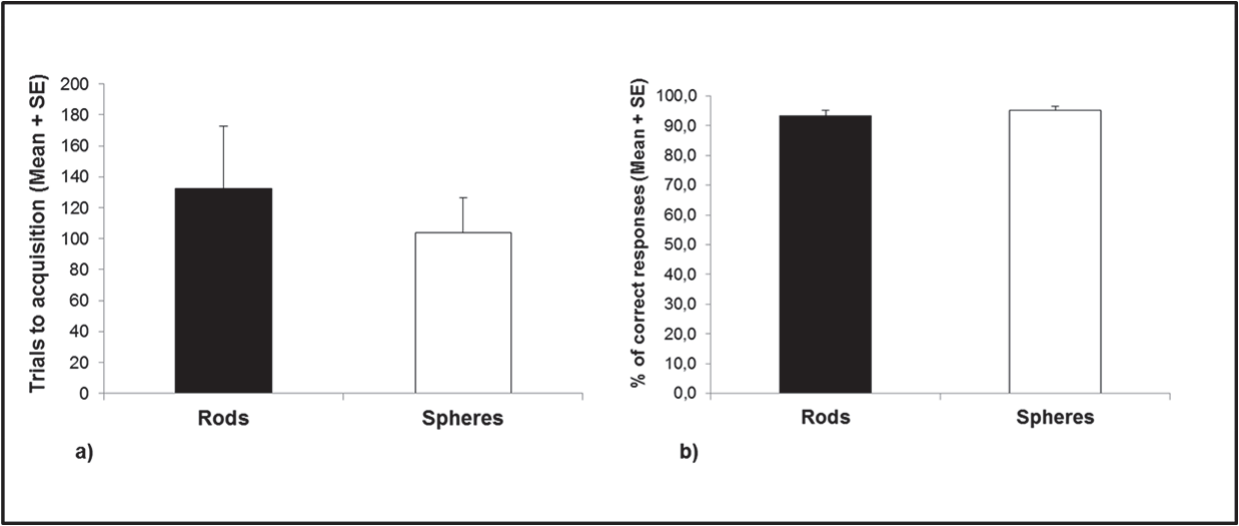
Monkeys’ performance in Experiment 2. Monkeys’ performance in the *Object* condition as a function of stimulus shape (rods and spheres) was reported considering: (a) the number of trials to achieve the learning criterion (mean ± SE); and (b) the mean percentage of correct responses in the last three training sessions (mean ± SE).

The difference between rods and spheres was not significant, neither for the number of trials to acquisition (Rods *M* = 132.6, *SE* = 40.2, Spheres *M* = 104.0, *SE* = 22.4, *F*_(1,5)_ = 0.42, *p* =. 547; Fig 4A) nor for the percentage of correct responses to acquisition (Rods *M* = 93.4, *SE* = 1.8, Spheres *M* = 95.2, *SE* = 1.4, *F*_(1,5)_ = 0.33, *p* =. 592; Fig 4B). Therefore, in the following analyses we collapsed all data for the different stimuli shapes (rods and spheres). Neither main effects nor interactions due to sex differences were significant.

### Number of trials

Capuchins required significantly fewer trials to achieve the learning criterion with foods than with objects [Foods: *M* = 34.8, *SE* = 4.3; Objects: *M* = 118.3, *SE* = 21.6; *t*(7) = -3.84, *p* =. 008]. Moreover, they required significantly fewer trials with objects than images [Images: *M* = 328.6, *SE* = 84.2; *t*(7) = -3.15, *p* =. 019]. Both these differences were still significant after Bonferroni correction for multiple tests (statistical significance was set at *p* =. 025).

### Accuracy scores

No significant differences were found in the mean percentage of correct responses to acquisition between objects and foods [Objects: *M* = 94.3%, *SE* = 1.0; Foods: *M* = 97.3%, *SE* = 0.7; *t*(7) = 2.10, *p* =. 082] and between objects and images [Images: *M* = 91.4%, *SE* = 1.2; *t*(7) = 2.50, *p* =. 046] (with Bonferroni correction for multiple tests, statistical significance was set at *p* =. 025).

### Transfer across tasks

Although all subjects received the *Object* condition as their third task, none of the subjects was able to achieve the learning criterion within the first three sessions (trial 1–48), neither for discriminating rods (Robot 71%, Pedro 75%, Robin Hood 67%, Sandokan 50%, Quincy 54%, Roberta 42%, Robiola 50%) nor for discriminating spheres (Robot 42%, Pedro 79%, Robin Hood 8%, Sandokan 92%, Quincy 29%, Roberta 54%, Robiola 50%). Even though one individual (Sandokan) showed an outstanding mean performance with the spheres within the first three sessions (92%), he did not achieve the criterion before the fourth session.

### Comparison between experimental and control groups

Experimental (Exp) and control (Ctrl) subjects were compared both in terms of learning speed and response accuracy to acquisition. The two measures considered were analysed by using a mixed model of ANOVA with Stimulus Shape (rods and spheres) as a within-subjects factor and Group (Exp and Ctrl) as a between-subjects factor. No significant main effects or interactions were found, neither concerning the number of trials to acquisition [Group: Exp, *M* = 118.29, *SE* = 21.64; Ctrl, *M* = 184.0, *SE* = 38.05; *F*_(1,9)_ = 2.67, *p* =. 137; Stimulus Shape: rods, *M* = 170.18, *SE* = 35.47; spheres, *M* = 114.18, *SE* = 16.08; *F*_(1,9)_ = 3.17, *p* =. 109; Group x Stimulus Shape: Exp rods, *M* = 132.57, *SE* = 40.22; Exp spheres, *M* = 104.0, *SE* = 22.36, Ctrl rods, *M* = 236.0, *SE* = 60.0; Ctrl spheres, *M* = 132.0, *SE* = 21.29; *F*_(1,9)_ = 1.03, *p* =. 337] nor concerning the accuracy to acquisition [Group: Exp, *M* = 94.35%, *SE* = 0.99; Ctrl, *M* = 93.23%, *SE* = 1.97; *F*_(1,9)_ = 0.33, *p* =. 581; Stimulus Shape: rods, *M* = 92.80%, *SE* = 1.39; spheres, *M* = 95.08%, *SE* = 1.23; *F*_(1,9)_ = 1.42, *p* =. 264; Group x Stimulus Shape: Exp rods, *M* = 93.45%, *SE* = 1.79; Exp spheres, *M* = 95.24%, *SE* = 1.42; Ctrl rods, *M* = 91.67%, *SE* = 2.40; Ctrl spheres, *M* = 94.79%, *SE* = 2.62; *F*_(1,9)_ = 0.11, *p* =. 753].

## Discussion

This study showed that tufted capuchin monkeys succeeded in learning to discriminate computer images, food items and objects of different sizes and that the presentation format significantly affected their learning speed and accuracy to acquisition. In keeping with our predictions, capuchins achieved the learning criterion faster with food stimuli compared to both images (Experiment 1) and objects (Experiment 2). They also required fewer trials to achieve the criterion with objects than with images (Experiment 2). Moreover, female capuchins showed higher levels of accuracy to acquisition with foods than with images (Experiment 1). Finally, we did not find evidence that capuchins immediately transferred the solution acquired in one task condition to other conditions (Experiment 1 and 2).

Capuchins’ ability to already perform accurately with food items after a few trials is consistent with results from previous studies on food choice tasks demonstrating that chimpanzees have a spontaneous powerful propensity to select food items on the basis of their size, preferring the larger items over the smaller ones [37, 39, 45, 76]. For example, Menzel [39] found that chimpanzees faced with arrays including four pieces of banana immediately selected them in order of size, with larger pieces being taken first. Additional studies also demonstrated that chimpanzees required to choose between different amounts of food can fail to select the larger amount because of biases towards the individual largest food items presented in the smaller option [37, 45]. Furthermore, in a quantity discrimination task Addessi et al. [42] showed that capuchins’ rate of correct choices was higher with food items than with tokens (i.e., objects with symbolic value) (but see also [77] for different results in baboons and macaques). Overall, these findings suggested that the ecological salience of food could affect choice behaviour in visual discrimination tasks. To maximise food intake during foraging activities, species might have evolved enhanced levels of attention towards—and/or strong propensity to detect—large (edible) items present in the visual field.

Besides the different salience of the stimuli, the type of procedure might also have played a significant role in at least three different ways, which are not mutually exclusive. First, the possibility to receive the chosen stimulus in the *Food* condition, where monkeys actually obtained what they chose, most resembles the ‘natural’ way of choosing and, as such, it might elicit improved judgement accuracy. Second, the possibility to directly manipulate food items after the choice might have facilitated subsequent size judgements in a highly manually skilled species like capuchin monkeys. Recent data on humans suggest that haptic information dominates in size discrimination processes in children younger than 8 years of age, i.e., when the integration of visual and haptic information is not fully developed [78]. Performance on visual size discrimination tasks was also found to be significantly worse in children of 9–16 years of age with movement disorders of the upper limbs (compared to age-matched typical children). This is possibly because, according to the cross-sensory calibration hypothesis, visual discrimination is impaired when the haptic sense is unavailable for manipulation and cannot be readily used to calibrate and fine-tune the visual experience of size [79]. Moreover, data on human adults showed that training subjects to discriminate shape categories by touch also improved their ability to visually discriminate the same stimuli and vice versa, indicating transfer of implicit knowledge across modalities [80]. Third, trials involving food items and objects forced monkeys to spend more time looking at the stimuli before they made a choice (i.e., the time needed to move the stimuli into the subject’s reach). Conversely, in the computerised procedure monkeys were able to choose as soon as the two stimuli appeared on the screen. Previous studies using computerised procedures have indicated that monkeys have a spontaneous tendency to inspect visual stimuli on a screen only for a very short time before taking a decision, and an increase of stimulus presentation time does not improve subjects’ discrimination performance [81]. Nevertheless, subjects’ response accuracy is positively affected by forcing them to actively attend to the stimulus, for example by repeatedly touching it to indicate a choice [12].

In the *Image* condition, capuchins required significantly more trials to achieve the criterion with the lines than with the circles. This difference between the shapes did not emerge when discriminating between food items (Experiment 1) or objects (Experiment 2). This finding indicates that some shapes require more training to be accurately discriminated than others, at least when stimuli are presented as two-dimensional images. From a perceptual point of view, 2D images carry less visual information compared to concrete 3D stimuli such as foods and objects, and this limitation could be more pronounced in certain shapes. Capuchins encountered more difficulties in discriminating different lines lengths, although their ratio (small/large = 0.56) was comparable to the ratio between the circles (small/large = 0.54). It is plausible to hypothesise that a decrease of the line length ratio might improve capuchins’ performance. Such a *distance effect* follows Weber’s law, according to which the discriminability of two magnitudes does not depend on their absolute magnitude differences but on their ratio, and has been reported in studies on line length discrimination using computer images with macaques [82] and crows [83]. Such an effect has also been reported for discrete quantity judgements in several animal species (e.g., ants [84]; birds [85]; fish [86]; monkeys [42, 87, 88]; apes [45, 89, 90]).

When learning speed was considered in terms of the number of days needed to achieve the learning criterion in Experiment 1 (i.e., total amount of time spent dealing with the tasks independently of the number of trials), performance in the *Food* and *Image* conditions no longer differed significantly, at least when circular-shaped stimuli were considered. This finding raises questions about memory mechanisms underlying learning performance such as: (1) the mere repetition of trials and (2) the neurophysiological time to generate an effective encoding, storage and retrieval of information in long-term memory. Although on the basis of our observations we can reasonably suppose that the increase of the number of trials may systematically improve subjects’ performance, we cannot completely exclude the possibility that time needed for memory consolidation partially acts independently of the number of trials that subjects receive in a daily experimental session. Other studies suggest that time and sleep have crucial impact on memory consolidation and, therefore, retrieval performance in great apes [91]. Further studies are needed to assess performance when the number of trials per day is equated for the *Image* and the *Food* condition.

No clear evidence emerged in support of capuchins’ ability to generalise size discrimination across different tasks. Capuchins did not immediately solve the discrimination problem when they received the images after foods and when they were presented with objects after both images and foods. An immediate high performance with food was observed in half of the individuals when food was presented either as the first or second condition, and this was probably due to this particular type of stimulus and its ecological relevance for the subjects. Thus, perceptual similarities among the tasks used in this study seemed insufficient to promote transfer processes. In other words, capuchins were obviously not capable of detecting functional similarities between the tasks, which prevented successful knowledge transfer. This is consistent with previous data, which showed that capuchins trained to solve an identity matching-to-sample problem with figures glued to vertical PVC boards failed to transfer their matching ability when confronted with figures of the same shape but presented as computer images on a touch-screen apparatus [16]. On the other hand, we know from past studies that capuchin monkeys trained to match stimuli according to rules are able to generalise these rules to novel stimuli when stimulus type (e.g., figures) and apparatus are identical [15, 16, 21, 22]. Capuchins also demonstrated that they recognise the correspondence between objects and their pictorial representations (i.e., photographs, silhouettes and line drawings) when presented simultaneously on the same apparatus [36]. Hence, in capuchin monkeys the transfer of knowledge and the recognition of correspondences in the visual modality seem to be possible, but only under certain circumstances and when crucial perceptual features in presentation format do not vary. At this point, it remains an open question as to what kind of feature similarity is required to enable a successful transfer, and which generalisation rules are preferentially internalised by capuchin monkeys.

The claim that the transfer of learned knowledge is mediated by the degree of perceptual similarity among stimulus environments dates back at least to the dawn of behaviourism and, more generally, to the study of learning principles [92]. However, exactly what constitutes perceptually salient features facilitating the transfer of knowledge across tasks has yet to be clearly established. Current knowledge concerning variations of behaviour in relation to changes of physical dimensions in the presentation format does not allow strong and cogent claims to be made. Among others, at least two aspects of the presentation format could constitute salient features potentially improving generalisation processes during transfer tests. First, the use of the same apparatus with an identical spatial arrangement of the stimuli could operate as a cue to indicate that a specific, familiar problem has to be solved even if novel stimuli are presented. Second, the use of the same type of stimuli, specifically 3D or 2D stimuli, should also avoid the need for object-picture correspondence abilities.

Overall, this study indicates that learning processes and transfer of knowledge in the visual domain strongly depend on task features, even in relatively simple discrimination problems. The same problem presented with different—although functionally equivalent—methods led to consistent variations in learning behaviour. Especially for manually skilled species, the possibility to exploit tactile feedback from the direct manual exploration of the stimuli seems to be crucial in order to calibrate and improve visual judgements. Taking the results and their implications together, one should be very cautious when comparing cognitive performances between different presentation formats, not to mention different experimental paradigms. Further studies are needed to clarify whether, and to what extent, visual discrimination may vary depending on the types of stimuli and/or different procedures and whether different animal species respond to procedural changes in similar ways or not.

## Supporting Information

S1 Table Individual data for Experiment 1. (DOCX)

S2 Table Individual data for Experiment 2. (DOCX)
