# Supplementary material for: Does Presentation Format Influence Visual Size Discrimination in Tufted Capuchin Monkeys (Sapajus spp.)?
Source: PLoS One. 2015 Apr 30;10(4):e0126001. doi: 10.1371/journal.pone.0126001 (PMC4416040; doi:10.1371/journal.pone.0126001)
Supplement: S1 Table — (DOCX) [file pone.0126001.s001.docx]

**S1 Table:** Individual data for Experiment 1

|  |  |  | **Foods** | | | | | | **Images** | | | | | |
| --- | --- | --- | --- | --- | --- | --- | --- | --- | --- | --- | --- | --- | --- | --- |
|  |  |  | **Wafers** | | | **Sticks** | | | **Circles** | | | **Lines** | | |
| **Subjects** | **Sex** | **Order** | **T** | **D** | **%** | **T** | **D** | **%** | **T** | **D** | **%** | **T** | **D** | **%** |
| Roberta | F | f/i | 48 | 6 | 100.0 | 48 | 6 | 95.8 | 104 | 4 | 87.5 | 448 | 14 | 87.5 |
| Robiola | F | f/i | 32 | 4 | 95.8 | 40 | 5 | 95.8 | 376 | 12 | 87.5 | 632 | 20 | 87.5 |
| Quincy | F | i/f | 56 | 7 | 100.0 | 48 | 6 | 95.8 | 120 | 4 | 91.7 | 440 | 14 | 87.5 |
| Rucola | F | i/f | 32 | 4 | 95.8 | 32 | 4 | 95.8 | 72 | 3 | 91.7 | 568 | 18 | 91.7 |
| Robot | M | f/i | 24 | 3 | 100.0 | 24 | 3 | 100.0 | 32 | 1 | 87.5 | 280 | 9 | 100.0 |
| Pedro | M | i/f | 32 | 4 | 95.8 | 24 | 3 | 91.7 | 88 | 3 | 87.5 | 232 | 9 | 95.8 |
| Robin H | M | f/i | 24 | 3 | 100.0 | 24 | 3 | 95.8 | 544 | 17 | 87.5 | 960 | 30 | 100.0 |
| Sandokan | M | i/f | 24 | 3 | 100.0 | 40 | 5 | 95.8 | 104 | 4 | 100.0 | 240 | 8 | 91.7 |

Note: T = number of trials to reach the learning criterion, D = number of days to reach the learning criterion, % = mean percentage of correct responses during the last three sessions of the training, F = female, M = male, f/i = food first/image second, i/f = image first/food second.
