# Supplementary material for: Does Presentation Format Influence Visual Size Discrimination in Tufted Capuchin Monkeys (Sapajus spp.)?
Source: PLoS One. 2015 Apr 30;10(4):e0126001. doi: 10.1371/journal.pone.0126001 (PMC4416040; doi:10.1371/journal.pone.0126001)
Supplement: S2 Table — (DOCX) [file pone.0126001.s002.docx]

**Table S2:** Individual data for Experiment 2

|  |  |  | **Objects** | | | |
| --- | --- | --- | --- | --- | --- | --- |
|  |  |  | **Spheres** | | **Rods** | |
| **Subjects** | **Sex** | **Group** | **T** | **%** | **T** | **%** |
| Roberta | F | E | 200 | 91.7 | 56 | 95.8 |
| Robiola | F | E | 80 | 95.8 | 344 | 87.5 |
| Quincy | F | E | 64 | 91.7 | 144 | 95.8 |
| Robot | M | E | 88 | 100.0 | 64 | 87.5 |
| Pedro | M | E | 96 | 95.8 | 40 | 100.0 |
| Robin H | M | E | 168 | 91.7 | 184 | 91.7 |
| Sandokan | M | E | 32 | 100.0 | 96 | 95.8 |
| Carlotta | F | C | 128 | 95.8 | 248 | 95.8 |
| Paprica | F | C | 184 | 87.5 | 264 | 87.5 |
| Paté | M | C | 136 | 100.0 | 360 | 87.5 |
| Vispo | M | C | 80 | 95.8 | 72 | 95.8 |

Note: T = number of trials to reach the learning criterion, % = mean percentage of correct responses during the last three sessions of the training, F = female, M = male, E = experimental, C = control.
